# Size-Dependent Deposition, Translocation, and Microglial Activation of Inhaled Silver Nanoparticles in the Rodent Nose and Brain

**DOI:** 10.1289/EHP234

**Published:** 2016-05-06

**Authors:** Esther Shin Patchin, Donald S. Anderson, Rona M. Silva, Dale L. Uyeminami, Grace M. Scott, Ting Guo, Laura S. Van Winkle, Kent E. Pinkerton

**Affiliations:** 1 Center for Health and the Environment, and; 2 Department of Chemistry, University of California, Davis, Davis, California, USA; 3 Department of Anatomy, Physiology and Cell Biology, School of Veterinary Medicine, University of California, Davis, Davis, California, USA; 4 Department of Pediatrics, School of Medicine, University of California, Davis, Sacramento, California, USA

## Abstract

**Background:**

Silver nanoparticles (AgNP) are present in personal, commercial, and industrial products, which are often aerosolized. Current understanding of the deposition, translocation, and health-related impacts of AgNP inhalation is limited.

**Objectives:**

We determined a) the deposition and retention of inhaled Ag in the nasal cavity from nose-only exposure; b) the timing for Ag translocation to and retention/clearance in the olfactory bulb (OB); and c) whether the presence of Ag in the OB affects microglial activity.

**Methods:**

Male Sprague-Dawley rats were exposed nose-only to citrate-buffered 20- or 110-nm AgNP (C20 or C110, respectively) or citrate buffer alone for 6 hr. The nasal cavity and OB were examined for the presence of Ag and for biological responses up to 56 days post-exposure (8 weeks).

**Results:**

The highest nasal Ag deposition was observed on Day 0 for both AgNP sizes. Inhalation of aerosolized C20 resulted in rapid translocation of Ag to the OB and in microglial activation at Days 0, 1, and 7. In contrast, inhalation of C110 resulted in a gradual but progressive transport of Ag to and retention in the OB, with a trend for microglial activation to variably be above control.

**Conclusions:**

The results of this study show that after rats experienced a 6-hr inhalation exposure to 20- and 110-nm AgNP at a single point in time, Ag deposition in the nose, the rate of translocation to the brain, and subsequent microglial activation in the OB differed depending on AgNP size and time since exposure.

**Citation:**

Patchin ES, Anderson DS, Silva RM, Uyeminami DL, Scott GM, Guo T, Van Winkle LS, Pinkerton KE. 2016. Size-dependent deposition, translocation, and microglial activation of inhaled silver nanoparticles in the rodent nose and brain. Environ Health Perspect 124:1870–1875; http://dx.doi.org/10.1289/EHP234

## Introduction

Silver nanoparticles (AgNP) are widely used for their antimicrobial activity and are found in textiles, cosmetics, food, and medical supplies. AgNP are currently being considered for use as anticancer agents (Wei et al. 2015). Approximately 50% of all nanoparticles in commercial use today are composed of silver (Project on Emerging Nanotechnologies 2015). Exposure to AgNP can occur through dermal absorption, oral ingestion, or inhalation. Inhalation of AgNP may occur during manufacturing, cloud seeding, or spraying for wound treatment, or through the use of AgNP as an aerosol disinfectant (Genter et al. 2012) or over-the-counter homeopathic spray for treatment of respiratory infections (Silver Lungs 2009; Silver Edge 2012). Because of their small size, AgNP can easily penetrate into the deep lung, which possesses an immense surface area for deposition, and can potentially transport to the systemic circulation. AgNP can also enter the nasal cavity and translocate to the brain via the olfactory epithelium, a direct connection between the nose and brain (Kovács 2004).

Previous studies have shown that nanoparticles, viruses, and molecules can bypass the blood–brain barrier and be transported from the olfactory epithelium in the nasal cavity to the olfactory bulb (OB) in the forebrain (Illum 2000; Mistry et al. 2009b). Three possible pathways have been suggested: *a*) transcellular transport across sustentacular cells of the olfactory epithelium, *b*) paracellular transport through junctions of the olfactory epithelium, or *c*) intracellular transport through axonal movement via olfactory nerve fascicles to the synaptic junctions within the OB (Illum 2000; Shepherd 1994). Nanoparticle translocation along axons of olfactory nerve fascicles and accumulation in the OB have been previously studied (Aschner 2009; De Lorenzo 1960; Elder et al. 2006, 2009; Hopkins et al. 2014; Oberdörster et al. 2004; Patel et al. 2011).

AgNP toxicity has been associated with the formation of Ag cations (Ag^+^), biochemically active agents (Brett 2006) that cause cytotoxic (Chernousova and Epple 2013; Loza et al. 2014; Xiu et al. 2012) and inflammatory responses (Wang et al. 2014) independent of the parent AgNP. The toxicity of Ag^+^ may be due to its interaction in biochemical processes with proteins, nucleic acids, and cell membranes (Chernousova and Epple 2013). Smaller (20 nm) AgNP were found to have faster dissolution rates and Ag^+^ formation than larger (110 nm) AgNP (Davidson et al. 2015; Wang et al. 2014).

Human health effects from AgNP exposure have been reported following dermal or oral administration (Gavanji et al. 2013); however, to the best of our knowledge, there are currently no published peer-reviewed studies that discuss the effects of AgNP inhalation in humans. Inhalation studies with animals demonstrated deposition of Ag in the lung and translocation of these nanoparticles to other organs (Anderson et al. 2015; Braakhuis et al. 2014; Genter et al. 2012; Ji et al. 2007; Sung et al. 2009). Toxic responses included epithelial erosion, proteinaceous exudates, and presence of inflammatory cells in the nasal cavity (Genter et al. 2012), airways and alveoli (Braakhuis et al. 2014; Sung et al. 2009), and consequent bile duct hyperplasia in the liver (Sung et al. 2009). The magnitude of these changes appears to be dependent on the dose or the size of the AgNP used, or on a combination of both (Braakhuis et al. 2014; Sung et al. 2009).

The objectives of this study were to determine *a*) the deposition and retention of Ag in the nasal cavity following inhalation exposure; *b*) the timing for Ag translocation and retention/clearance in the OB; and *c*) whether the presence of Ag in the OB affects the activity of microglial cells, the resident macrophages of the OB and the brain. Nasal cavities and OBs in this study were obtained from the same animals used in the study by Anderson et al. (2015), in which a single acute inhalation dose of 20- or 110-nm AgNP (C20 or C110, respectively) was delivered over a period of 6 hr, and biological samples were obtained immediately after exposure (T0) and at 1 (T1), 7 (T7), 21 (T21), and 56 (T56) days post-exposure.

## Methods

### Particles

AgNP [20 or 110 nm in diameter (nanoComposix, Inc., San Diego, CA)] were suspended in citrate buffer (Fisher Scientific, Pittsburgh, PA). The AgNP were procured, characterized, and supplied by the National Institute of Environmental Health Sciences (NIEHS) Centers for Nanotechnology Health Implications Research (NCNHIR) Consortium [National Institutes of Health (NIH) 2015]. Citrate stabilized the particles by creating electrostatic repulsion, thus preventing AgNP aggregation and enabling control of the final particle size (Tolaymat et al. 2010). Physicochemical characterization of the AgNP has been described by Anderson et al. (2015), Silva et al. (2015), and Wang et al. (2014).

### Animals

Ten- to 12-week-old male Sprague Dawley rats (Harlan Laboratories, Livermore, CA) were used to maintain consistency with other studies (Anderson et al. 2015; Ji et al. 2007; Silva et al. 2015; Sung et al. 2009). Upon arrival, rats were randomly assigned to one of three groups: *a*) C20-, *b*) C110-, or *c*) citrate control–treated (*n* = 8 per AgNP treatment group per time point, and *n* = 8 for citrate control). The rats were allowed to acclimate for 1 week before AgNP exposure and were housed two per cage and given access *ad libitum* to water and a standard laboratory rodent diet (Purina Mills, St. Louis, MO) except during exposure periods. Animals were handled under protocols in accordance with the Guide for the Care and Use of Laboratory Animals (National Research Council 2011) and the Institutional Animal Care and Use Committee of the University of California (UC), Davis.

### Aerosolization of AgNP

Rats were acclimated in inhalation exposure tubes (Teague Enterprises, Woodland, CA) for 1 week prior to the scheduled exposure to simulate exposure conditions. Animals were trained to enter and remain in the exposure tubes until released. Time in the tube increased up to 6 hr at a gradual rate over the training week. This training served to minimize/prevent confinement stress during the actual AgNP aerosol exposure period.

Rats were exposed via nose-only inhalation to C20, C110, or citrate buffer for 6 hr. The aerosolization regimen has been previously described (Anderson et al. 2015). Briefly, a 6-jet collision nebulizer (Mesa Labs, Waltham, MA) was used to aerosolize the AgNP suspensions into fine droplets. Water was removed from the droplets using two TSI diffusion dryers (TSI, Shoreview, MN), and particle charge was neutralized with a Krypton-85 source. Samples were collected throughout the exposure period to characterize the aerosol with gravimetric, X-ray fluorescence (XRF), cascade impactor, transmission electron microscope (TEM), and real-time size mobility particle scanner (SMPS) measurements, as previously described by Anderson et al. (2015). Airborne particle number was also calculated based on the airborne concentration of Ag collected by XRF analysis. Estimated Ag deposition in the nose normalized to surface area of the rat nasal epithelium was 4 μg/cm^2^ or 1 μg/cm^2^ after exposure to aerosolized suspensions of C20 or C110, respectively. This dose was selected to approximate human exposure after 1 day of light work in a worst-case occupational scenario, based on assumptions and calculations described in detail in Supplemental Material, “Relating experimental rodent inhalation of AgNP to human occupational exposure.”

### Necropsy and Tissue Collection

Biological samples were collected at all post-exposure time points. All rats were anesthetized by intraperitoneal injection of sodium pentobarbital (120 mg/kg) and euthanized via exsanguination. The nasal epithelia and OBs from five animals per group were collected and frozen in liquid nitrogen for silver detection via inductively coupled plasma mass spectrometry (ICP-MS). In addition, the OBs from three rats per group were fixed in 4% paraformaldehyde for silver detection via autometallography, and sections of these fixed OBs were also used for microglial examination and cytokine expression assays.

### Preparation of Tissue Sections

To detect silver by ICP-MS, the entire animal head was cut sagittally to expose the nasal epithelium. All the strippable epithelium, including the septal and turbinate walls, was collected to maintain collection consistency between rats.

The nasal epithelia and whole OBs were placed into liquid nitrogen in 15-mL conical tubes. Frozen tissues were lyophilized using LabconcoFreeZone 2.5 (Kansas City, MO) for 48 hr, after which tissue weights were determined. The tissues were subsequently digested using equal parts of 70% trace metal–grade nitric acid (Fisher Scientific, Waltham, MA) and 30% hydrogen peroxide (EMD Millipore, Billerica, MA). Digestion in nitric acid proceeded at 70°C for 2 hr, and then the samples were cooled to room temperature (25°C) for 1 hr. Subsequent digestion in an equal volume of hydrogen peroxide proceeded at 70°C for 15 hr followed by cooling to room temperature. All samples were brought to a known volume and diluted 5:1 with Milli-Q® water.

Samples were analyzed at the UC Davis Interdisciplinary Center for Plasma Mass Spectrometry using an Agilent 7500CE ICP-MS (Agilent Technologies, Palo Alto, CA). Silver concentration (micrograms Ag/gram tissue) was calculated from the known tissue weight (grams), sample volume (milliliters), and measured Ag content (nanograms/milliliter). The mean and standard error of the silver concentration were calculated, and statistical differences were compared between C20- or C110-treated and citrate control groups at each time point. The hypothetical number of AgNP in the nose and in the OB was also estimated from the mass of Ag detected by ICP-MS and normalized to known tissue weight. For these calculations, we assumed that the AgNP maintained a spherical shape with no dissolution throughout the post-exposure period.

For silver detection via autometallography, nasal sections were deparaffinized with toluene, hydrated in decreasing concentrations of ethanol, and stained with equal volumes of developer and enhancer from a silver enhancement kit for light and electron microscopy (Ted Pella Inc., Redding, CA) for 15 min. Sections were then dehydrated in increasing concentrations of ethanol and toluene and coverslipped with ClearMount™ permanent mounting medium (Thermo Fisher Scientific). All images were collected using a Zeiss AxioLab.A1 microscope.

### Microglial Activation and TNF-*α* Detection in the Olfactory Bulb

For microglial examination and cytokine expression, OBs were fixed in 4% paraformaldehyde for 7 days and embedded in paraffin in a sagittal orientation. Five-micrometer-thick sections were cut, deparaffinized with toluene, hydrated in decreasing concentrations of ethanol, and rinsed with phosphate-buffered saline with Tween (PBST; Sigma Aldrich, St. Louis, MO) before antigen retrieval. Slides were immersed in citric acid (Thermo Fisher Scientific; pH = 6) for 30 sec at 125°C for antigen retrieval, followed by 10 sec at 85°C. Sections were then cooled for 15 min, rinsed with PBST for 3 min and with 3% hydrogen peroxide for 5 min, washed in PBST three times at 3 min each; the PBST washes were followed by a nonspecific block, Protein Block (Dako, Carpinteria, CA), for 30 min at 20°C. The sections were then incubated with the primary antibody, anti-ionized calcium-binding adapter molecule 1 (anti-Iba1; Abcam Inc., Cambridge, MA), for 3 hr to visualize microglial cells in a state of rest or activation or with anti-tumor necrosis factor alpha (anti-TNF-α; Abcam Inc.) for 1 hr at 20°C. Sections were washed with PBST three times for 3 min each, then incubated in secondary antibody, biotinylated affinity-purified goat anti-rabbit immunoglobulin G (IgG; Vector Laboratories, Burlingame, CA), for 1 hr at 20°C or in horseradish peroxidase (HRP)-labeled polymer (rabbit) (Dako) for 30 min at 20°C. The sections were again washed with PBST for 5 min, incubated with Avidin/Biotin Complex (Vector Laboratories) for 30 min at 20°C, and rinsed with PBST again three times for 5 min each. The sections were then incubated with 3,3′-diaminobenzidine (DAB) and substrate (Dako) for 5 min, counterstained with Harris’s Hematoxylin (MasterTech, Inc., Lodi, CA), and coverslipped with ClearMount™ permanent mounting medium (Thermo Fisher Scientific).

**Figure 1:**
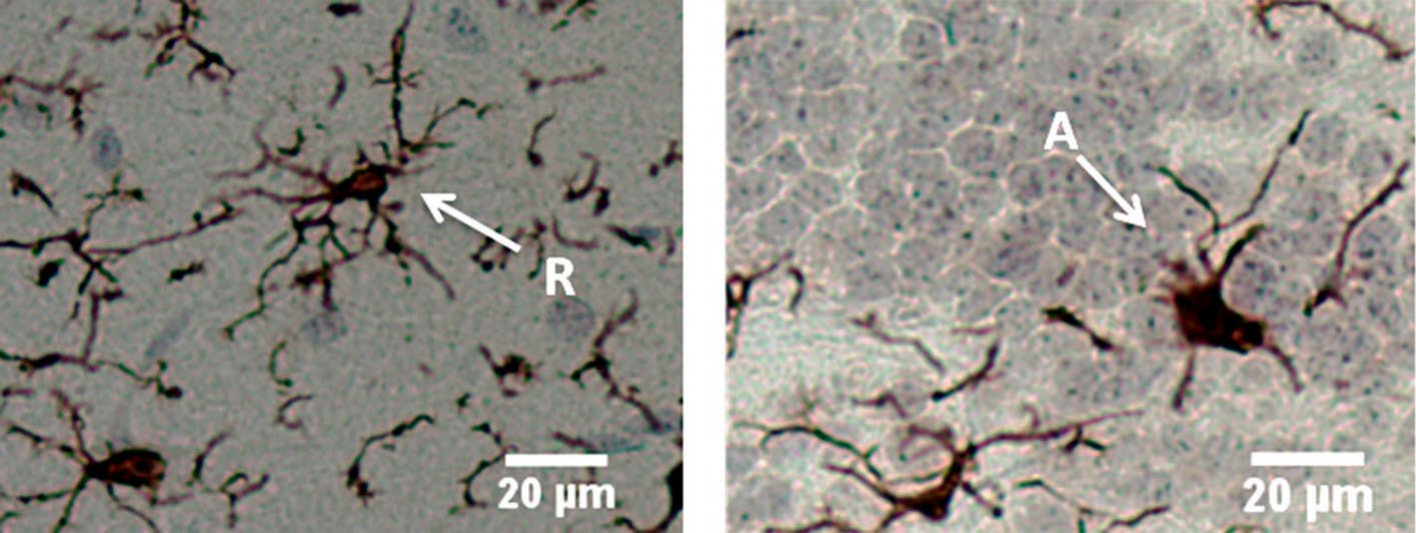
Microglial morphology of rat olfactory bulb stained with anti-Iba1 antibody. Left, resting/ramified (R) microglia exhibiting thin, highly branched protrusions extending from the cell body; right, activated microglia (A) exhibiting amoeboid morphology with shorter, stouter processes. Left, rat treated with 110-nm silver nanoparticles 21 days post-exposure; right, rat treated with 20-nm silver nanoparticles 21 days post-exposure. Bar = 20 μm.

Microglial morphology was observed to determine whether they were resting or activated. Briefly, resting microglia were defined as cells with at least two highly branched (ramified) processes extending at least twice the length of a highly elliptical nucleus. Activated microglia were defined as cells with significantly shortened branching processes extending from a slightly enlarged cell nucleus (Figure 1). Microglia were counted in 10 randomly sampled, nonoverlapping fields per histological section per animal and were observed at an objective magnification of 20× using a Zeiss AxioLab.A1 microscope. Resting and activated microglia were also counted in identical histological fields for each animal to a total of 50 cells/animal, and the ratio of active to total cells was calculated. The mean value and percent difference (compared with control) were determined for each exposure group at each time point. The incidence of TNF-α staining in the OB was measured using ImageJ (Rasband 1997–2016), and images were acquired with a 5× objective on the same Zeiss microscope. The intensity of TNF-α staining within total areas of expression was determined for each exposure group at each time point. Three animals per AgNP treatment group per time point were examined along with six control animals.

### Silver Transport Analysis

The percentage of Ag translocated to the OB via the olfactory region of the nasal cavity at T0 for C20 and at T56 for C110 was calculated. Based upon the computational fluid dynamics model proposed by Garcia and Kimbell (2009), ~24.55% and ~35% of C20 and C110 in the nose, respectively, deposited in the olfactory region. These percentages were then used with our data for total Ag deposition in the nasal cavity at T0 for C20 and at T56 for C110 to determine how much of the total Ag deposition was deposited only in the olfactory region. The percentage of Ag translocation was then calculated from the original total Ag deposited in the olfactory region of the nasal cavity.

### Statistical Analysis

All of the data presented here were analyzed using JMP 10.0.0 statistical software (SAS Institute Inc., Cary, NC). No outliers were identified, with outliers being quantified as how far the value was from the others using Grubbs’ test. The ICP-MS data were first tested for deviations from the normality and homoscedasticity (equal variance) assumptions of analysis of variance (ANOVA) using Shapiro–Wilk (on model residuals) and Levene tests, respectively. To achieve normal distributions, data were log-transformed (log_10_). ANOVA and post hoc Tukey HSD tests were performed for all of the ICP-MS and particle number data to determine specific significant differences between treatment groups. These tests were performed using a significance level of *p* < 0.05. Significance for microglial activation was determined by ANOVA and least significant difference (LSD) Student’s *t*-test to determine significant differences (*p* < 0.05) at each time point resulting from exposure type (C20 or C110 vs. control).

## Results

### Particle Characterization and Aerosolization

AgNP characteristics were determined by Anderson et al. (2015) and are summarized in Table 1. Anderson et al. (2015) observed minimal changes in the hydrodynamic diameter of AgNP determined by dynamic light scattering (DLS) in suspension before (from sealed containers) and after exposure (from the nebulizer). Particle mass concentration from the generated aerosols was determined gravimetrically. In addition, airborne particle number concentrations were estimated using the XRF-determined silver mass concentrations (Anderson et al. 2015) (Table 1).

**Table 1:** Characterization of 20- and 110-nm silver nanoparticles (C20 and C110, respectively) in suspension and following aerosolization (adapted from Anderson et al. 2015).

Parameter	Method	C20	C110
Hydrodynamic diameter before exposure (nm)^*a*^	Dynamic light scattering (DLS)	27.06 ± 0.15	111.2 ± 0.2
Hydrodynamic diameter after exposure (nm)^*a*^	Dynamic light scattering	27.24 ± 0.21	106.6 ± 0.2
Geometric mean size [nm (standard deviation)]	Size mobility particle scanning	77.4 (1.8)	78.2 (1.8)
Total particle mass concentration (mg/m^3^)	Gravimetric analysis	13.9 ± 2.3	12.4 ± 2.5
Airborne silver ion concentration (mg/m^3^)	X-ray fluorescence	7.2 ± 0.8	5.3 ± 1.0
Airborne particle number based on airborne concentration of silver (number/m^3^)^*b*^	Calculated	1.63 × 10^14^	7.24 × 10^11^
^***a***^Hydrodynamic diameter of particles was measured from sealed containers before exposure and from particles recovered from the nebulizer at the end of exposure. Dynamic light scattering was performed using a Zetasizer Nanosizer ZEN 1690. ^***b***^Airborne particle number = Airborne concentration from X-ray fluorescence (milligrams/cubic meter)/[mass of C20 or C110 (grams) × 1,000].			

### Silver Content in the Nasal Epithelium and OB

**Figure 2:**
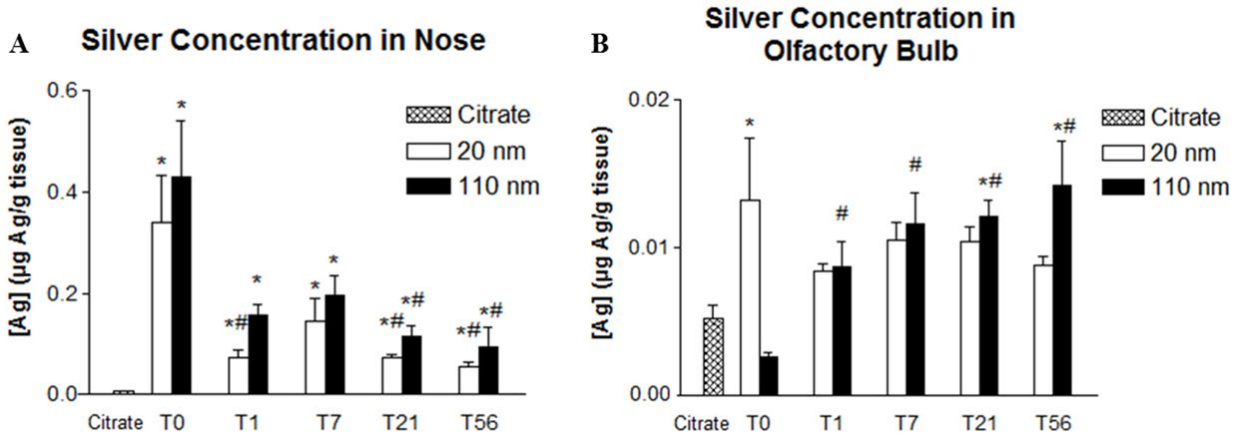
Comparison of silver concentration in the nose (A) and olfactory bulb (B) between citrate (control) and 20- and 110-nm silver nanoparticles (AgNP) at different post-exposure days. *Significantly different from citrate control (*p* < 0.05). ^#^Significantly different from the mean value at T0 in animals that had the same exposure (*p* < 0.05). Values are the mean ± SE. The level of detection is 0.011 μg Ag/g tissue. The mean value was obtained from 5 animals per particle type and time point.

The limit of detection (LOD) was 11 ppb, with all data reported as measured values without regard to the LOD. Silver concentrations in the nasal epithelia of rats exposed to both C20 and C110 were significantly higher than those in controls at all time points post-exposure (Figure 2A). For both particle sizes, concentrations were highest immediately after treatment (T0) and lowest at T56, with significantly lower concentrations relative to T0 for C20 at T1, T21, and T56 and for C110 at T21 and T56. Although some data points are below the detection limit (citrate buffer, C20 at T1–T56, and C110 at T0–T1), if viewed as measured, the trends show that after exposure to C20 (vs. control), ~4% (0.013 μg/g in the OB at T0 after C20 exposure vs. 0.34 μg/g in the nasal epithelium at T0 after C20 exposure) of the Ag was translocated from the nose to the OB, and the concentration of silver detected in the OB at T0 (0.013 ± 0.0042) was significantly higher than the concentration in controls (Figure 2B). At subsequent time points after C20 exposure, the concentration of silver in the OB of treated animals was not significantly different from background levels in controls. In contrast, the concentration of silver in the OB was significantly higher than the T0 concentration in C110-exposed animals at all subsequent time points (Figure 2B). Silver detection via autometallography showed very sparse amounts only in C20-exposed animals at T0. Other levels and time points failed to clearly show Ag ions or particles.

### Microglial Activation and Cytokine Expression

**Figure 3:**
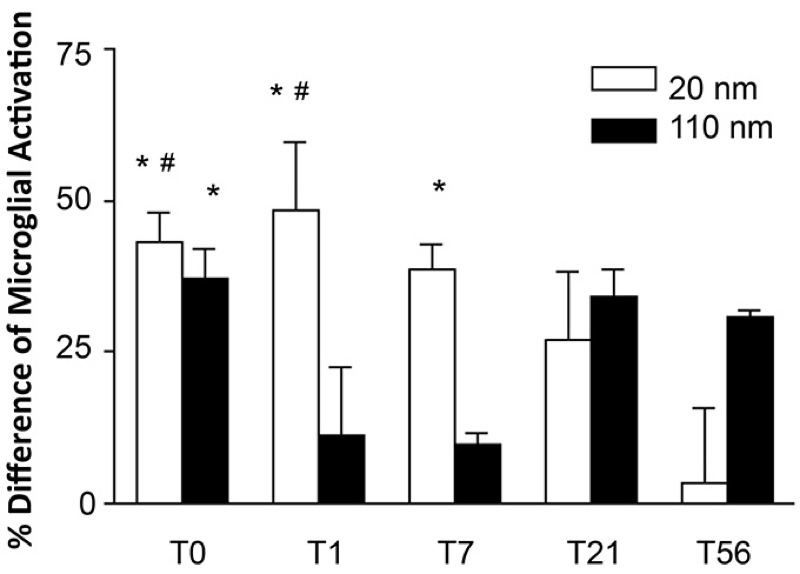
Comparison of percent difference from control of activated microglia in the olfactory bulb between 20- and 110-nm silver nanoparticles at different post-exposure days. *Significantly different from citrate control (*p* < 0.05). ^#^Significantly different from the mean value at post-exposure day 56 (T56) in animals that had the same exposure (*p* < 0.05). Values are the mean ± SE. The mean value was obtained from 3 animals per particle type and time point except for the group treated with 110-nm silver nanoparticles at T56, where the mean value was obtained from 2 animals.

**Figure 4:**
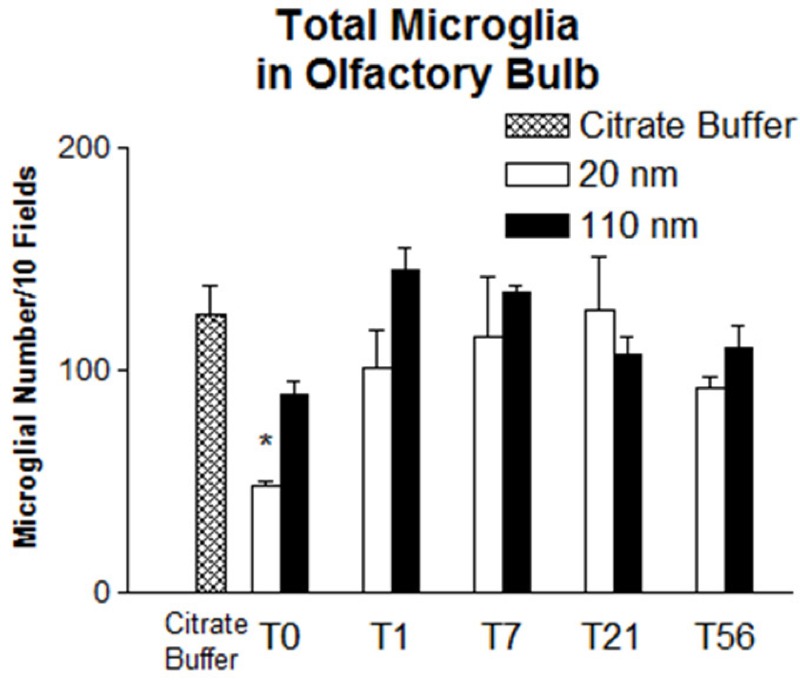
Comparison of microglial number/10 fields in the olfactory bulb between controls and rats exposed to silver nanoparticles at different post-exposure days. Values are the mean ± SE. The mean value was obtained from 3 animals per particle type and time point except for the group treated with 110-nm silver nanoparticles at post-exposure day 56, where the mean value was obtained from 2 animals.

Microglial morphology in the OB was observed and differentiated between resting and activated states in order to determine whether the two AgNP types elicited responses (Figure 1). Compared with controls, proportions of activated microglia in the OB were significantly higher among C20-treated animals at T0, T1, and T7, and in C110-treated animals at T0 (see Table S1 for values in individual animals and Figure 3 for mean values). The mean proportion of activated microglia after C20 exposure decreased after T1 such that proportions were not significantly different from control animals at T21 and T56 and were significantly higher at T0 and T1 than at T56, when the mean proportion of activated microglia was only 3% higher (± 12%) than the mean in controls. Conversely, C110 produced a variable pattern, with minimal activation at T1 and T7 (11% ± 11% and 10% ± 2%, respectively), in contrast to a relatively high degree of microglial activation at T0, T21, and T56 (37% ± 5%, 34% ± 5%, and 31% ± 1%, respectively) with significance shown only at T0 (Figure 3). The mean microglial number/field was significantly lower than in controls for C20 animals at T0, suggesting a transient cytotoxic response to nanoparticles in the OB (Figure 4). Although the mean microglial number/field at T0 was lower in C110 animals than in controls, the difference was not significant, and there were no significant differences from controls at any other remaining time point for either AgNP exposure group.

TNF-α staining, although observed in the OB, was not found to be significantly different from that of the controls at any time point for either treatment group (data not shown).

### Silver Transport to the Olfactory Bulb via the Olfactory Region

The amount of Ag deposited in the olfactory region of the nose was 0.08 μg Ag/g tissue and 0.15 μg Ag/g tissue for C20 and C110, respectively. The percentages of silver translocated to the OB from the olfactory region of the nose were 16% and 9% at T0 for C20 and at T56 for C110, respectively.

## Discussion

Short-term inhalation of AgNP resulted in the accumulation of silver in the OB over time, with some differences according to nanoparticle size. Following a single 6-hr inhalation exposure, C20 and C110 AgNP were deposited and taken up in the epithelium of the nose, where they were retained and subsequently transported to the OB. Silver content in the nose and OB was monitored for up to 56 days (8 weeks) post-exposure and correlated to varying degrees to microglial responses in the OB. These findings suggest that *a*) AgNP size does not affect retention/clearance of Ag in the nose or OB; *b*) C20 AgNP readily translocate to the OB, where they elicit an immediate response; and *c*) C110 AgNP accumulate and elicit a varied response in the OB immediately after exposure and at later time points.

Irrespective of the original particle size, Ag concentrations in the nose remained significantly higher than in controls ≤ 8 weeks post-inhalation. We believe that this persistence is most likely due to interstitial and/or intraepithelial localization, as was previously noted in the lungs of animals exposed to the same treatments (Anderson et al. 2015). However, in the present study, autometallography failed to conclusively demonstrate the precise localization of Ag in the nose, perhaps owing to the relatively small amounts of Ag remaining there compared with those in the lung. Ag in the nasal cavity can be cleared from the respiratory tract via several pathways: *a*) mucocilliary movement to the nasopharynx and into the gastrointestinal tract; *b*) translocation to the circulatory system; *c*) translocation to the lymphatic system; and *d*) translocation to the OB. We observed significant Ag in the OB at T0 for C20 and at T21 and T56 for C110 compared with controls, suggesting that Ag is transported from the nose to the OB.

Previous studies have reported transport of nanoparticles to the OB via the olfactory system in the nose (Elder et al. 2009; Hopkins et al. 2014; Oberdörster et al. 2004) as well as higher levels of Ag in the OB than in the rest of the brain as measured by atomic absorption spectroscopy (Ji et al. 2007; Sung et al. 2009). Genter et al. (2012) visualized Ag in the OB and in cells lining the lateral ventricle via autometallography following intranasal administration and surmised that high Ag deposition in the OB, which is located closer to the inhalation site than the rest of the brain, suggests that Ag particles reach the brain via the nose. The investigators showed aggregation of Ag in the turbinated areas of the nasal cavity lined with olfactory epithelium, which is one of the main direct portals of entry to the OB (Genter et al. 2012).

Garcia and Kimbell (2009) reported that even though total nasal deposition (of inhaled 1- to 100-nm particles) decreased with increasing particle size, a higher proportion of larger nanoparticles deposited in the olfactory region than in other regions (i.e., respiratory, squamous, and transitional epithelium) of the nasal cavity. Using the computational fluid dynamics model proposed by Garcia and Kimbell (2009), we estimated that olfactory deposition of C20 and C110 would be ~25% and ~35% of the total nasal deposition, respectively. Based on this information and on known Ag concentration determined by ICP-MS, ~16% of C20 was translocated by T0 and ~9% of C110 was translocated by T56 from the olfactory region of the nose to the OB of the brain (see Table S2). These estimates are similar to those reported by Oberdörster et al. (2004), who showed consistent accumulation of 36-nm ^13^C (carbon particles) and approximately 20% translocation from the olfactory region to the OB. In the study by Oberdörster et al., axonal transport via the olfactory sensory neurons was the main translocation pathway over the 7-day post-exposure period.

We believe that the physicochemical properties (size and dissolution rate) of C20 and C110 are most likely responsible for their different deposition in the OB over time. Although C20 AgNP are small enough to be rapidly transported through the olfactory axons, C110 AgNP are not. Mistry et al. (2009a) showed that 100-nm polystyrene nanoparticles could not translocate from the olfactory axon, which tapers off through the basement membrane, to the OB in mice. Based on a previous publication reporting that rabbit olfactory axons have an average diameter of ~200 nm and that many axons have diameters < 100 nm (De Lorenzo 1960; Mistry et al. 2009b), we surmise that the average diameter of rat olfactory sensory neurons is likely ≤ 100 nm. Although it is unknown whether Ag present in the OB is ionic or particulate, we speculate that Ag from C110 did not travel from the nose to the OB by olfactory sensory neuron transport because solid 110-nm particles are unlikely to “fit” in the axons. However, if the diameter of rat olfactory axons is > 100 nm, it may be possible for C110 AgNP to transport (slow anterograde) from the nose to the OB. In this case, transport favors dissolved Ag ions or smaller AgNP formed after the initial dissolution event.

Differences in OB Ag content at various time points after exposure to C20 (vs. C110) may be attributable to the ability of C20 to directly transport along the axons and to dissolve relatively quickly. Rapid ion dissolution is enabled by the presence of organic molecules (Loza et al. 2014) and by oxidation (Liu et al. 2012). Mucin in the nose consists of carbohydrates and heavily glycosylated proteins rich in serine and threonine residues (Thornton et al. 2008), which can aid in particle dissolution, and the nose is a continuous site of oxidation. Particle size can also contribute to dissolution, with smaller citrate-coated C20 undergoing faster dissolution than larger C110 (Wang et al. 2014; Zhang et al. 2011). Whereas C20 may immediately dissolve to form Ag^+^ and, in subsequent ionic reactions, form smaller AgNP, C110 sheds continuously, as evidenced by concurrent high concentrations of Ag^+^ around the parent AgNP over time (Davidson et al. 2015). C20 could be rapidly transported to the OB with the highest deposition occurring at the earliest time point (T0). In contrast, owing to a slower dissolution process, C110 may not reach the OB until approximately 21 days after exposure because C110 produces more Ag^+^ ions that continuously go through cycles of small-particle formation and dissolution until the parent AgNP are depleted (Davidson et al. 2015). Thus, the time for transport and accumulation of C110 Ag in the OB is extended.

Previous studies confirmed the presence of Ag in the OB with no observable histopathology (Genter et al 2012; Ji et al. 2007; Sung et al 2009). Only Genter et al. (2012) examined microglial activation resulting from Ag exposure: three groups of mice were exposed to sterile water or to different doses of 25-nm AgNP and were sacrificed at 1 or 7 day(s) after a single intranasal instillation. Despite detection of Ag in the OB, no inflammatory cell infiltrates or microglial activation was observed. The Genter et al. (2012) study and our study differ by AgNP coating. Whereas we coated the AgNP with citrate to prevent aggregation, Genter et al. (2012) did not and observed (by DLS) agglomeration of the 25-nm AgNP into 118-nm aggregates. This aggregation could have hindered the release of Ag^+^ for > 7 days, thereby preventing the observation of microglial activation. Indeed, the present study suggests that larger (C110) AgNP do not produce a significant amount of Ag^+^ immediately following deposition given that a significant amount of silver in the OB was only found 21 days post-exposure.

In this study, microglial activation was observed as an initial response. Although microglial activation can be beneficial to cellular maintenance and clearance of foreign substances, if prolonged and over-stimulated, this process can be detrimental owing to the release of toxic factors, such as free radicals, and to the potentiation of neuronal loss and damage (Block et al. 2007). After exposure to C20, animals had a significantly lower mean total microglial number than that in controls (T0; Figure 4), and the proportion of activated microglial cells was significantly higher than in controls at T0 and T1, but it was comparable to controls by T56 (Figure 3). Exposure to C110 produced the highest microglial activation at T0 that then declined by T7 and increased again (nonsignificantly) at T21 and T56. However, no significant differences in TNF-α staining were noted between treatment and control groups at any time point, suggesting the lack of a robust proinflammatory cytokine response during microglial activation in this study.

The microglial activation observed here may be caused by Ag^+^. A correlation has been noted between toxicity and dissolution of Ag^+^ from AgNP *in vitro* (Brett 2006; Chernousova and Epple 2013; Wang et al. 2014). The faster dissolution rate of C20 could explain the higher activation of microglia at earlier (vs. later) time points (T0 and T1). Discrepancies between the amount of Ag deposition and microglial activation after exposure to C110 may be explained by a slower Ag^+^ production rate by the larger AgNP. Although the presence of Ag in the OB may have been too low to detect at T0, trace amounts of Ag^+^ could cause microglial activation. By T1, Ag^+^ could also reduce and reform into small AgNP, making Ag^+^ unavailable to elicit a response. Because AgNP continuously shed Ag^+^ until their depletion, T56 could be the time when these Ag^+^ ions are most present, thus causing higher microglial activation at this time point. A previous study by Söderstjerna et al. (2014) showed microglial activation in cultured neural retina explants resulting from exposure to 20- or 80-nm AgNP at final concentrations of 0.0035 and 0.22 μg/mL, respectively. Although 80-nm AgNP produced significant numbers of activated microglia compared with controls, 20-nm AgNP produced a nonsignificant elevation. To the best of our knowledge, there are no other reports demonstrating microglial activation by AgNP.

## Conclusion

This study is the first to demonstrate the ability of Ag (from two different-sized AgNP)to be deposited, translocated, and retained from the nose to the brain (OB) at levels sufficient to cause microglial activation for up to 56 days post-exposure following a single 6-hr inhalation period. The long-term retention of measurable Ag in the nasal cavity and the OB is also an important finding. The mechanism of microglial activation, the physicochemical state of Ag in tissues over time, and the potential to elicit neurological effects following inhalation of differently sized AgNP clearly merits further investigation. The present study provides useful information to increase awareness about the possible importance of regulating inhaled AgNP in occupational settings, and it demonstrates potential risks associated with using aerosolized AgNP as holistic therapeutic or antimicrobial agents.

## Supplemental Material

(326 KB) PDF
