# Supplementary material for: Size-Dependent Deposition, Translocation, and Microglial Activation of Inhaled Silver Nanoparticles in the Rodent Nose and Brain
Source: Environ Health Perspect. 2016 May 6;124(12):1870–5. doi: 10.1289/EHP234 (PMC5132640; doi:10.1289/EHP234)
Supplement: (326 KB) PDF [file EHP234.s001.acco.pdf]

Environ Health Perspect
DOI: 10.1289/EHP234

Note to readers with disabilities: *EHP* strives to ensure that all journal content is accessible to all readers. However, some figures and Supplemental Material published in *EHP* articles may not conform to 508 standards due to the complexity of the information being presented. If you need assistance accessing journal content, please contact ehp508@niehs.nih.gov. Our staff will work with you to assess and meet your accessibility needs within 3 working days.

Supplemental Material

Size-Dependent Deposition, Translocation, and Microglial Activation of Inhaled Silver Nanoparticles in the Rodent Nose and Brain

Esther Shin Patchin, Donald S. Anderson, Rona M. Silva, Dale L. Uyeminami, Grace M. Scott, Ting Guo, Laura S. Van Winkle, and Kent E. Pinkerton

Table of Contents

Relating experimental rodent inhalation of AgNP to human occupational exposure

Table S1. Data on numbers of activated microglia to total microglia in control and AgNP treated animals

Table S2. Calculations of highest silver transport from the nose to the olfactory bulb at T0 for 20 nm and at T56 for 110 nm

References

Relating experimental rodent inhalation of AgNP to human occupational exposure

To derive the experimental conditions of rodent inhalation exposure used in this study to the equivalent occupational exposure conditions for AgNP, the following assumptions and calculations were made:

1. AgNP deposition (mg) at a peak occupational area concentration (C) of 0.289 mg/m^3 with particles ranging 0.015-710.5 nm in aerodynamic diameter (Lee et al. 2012)

Assumptions:

- F = 44% = maximum fraction of AgNP deposited in the nasal region (Cheng and Swift 1995)
- T = 9600 min = monthly exposure period (60 min/h, 8 h/day, 5 days/wk, 4 wks)
- V = 15 L/min = minute ventilation for an adult human (Cheng and Swift 1995)

Calculation of monthly AgNP deposition in the human nose:

$$44\% \times \frac{0.289 \text{ mg Ag NP}}{\text{m}^3} \times \frac{\text{m}^3}{1000 \text{ L}} \times 9600 \text{ min} \times \frac{15 \text{ L}}{\text{min}} = 18.31 \text{ mg AgNP}$$

2. Monthly AgNP deposition normalized to surface area of the human nasal cavity (HNC)

Assumption:

- 181 cm^2 = SA of human nasal cavity (HNC) (Derelanko and Auletta 2014)

Calculation:

$$\frac{18.31 \text{ mg Ag NP}}{181 \text{ cm}^2 \text{ HNC}} = \frac{0.10 \text{ mg Ag NP}}{\text{cm}^2 \text{ HNC}}$$

3. Estimated rat exposure to AgNP in the current experiment

Assumptions:

- F_{30} = 11% = fraction of 30 nm AgNP deposited in the nasal region (Cheng and Swift 1995)
- F_{110} = 4% = fraction of 110 nm AgNP deposited in the nasal region (Cheng and Swift 1995)
- C_{30} = 7.2 ± 0.8 mg/m^3 = airborne concentration of silver in the 20 nm AgNP aerosol (Anderson et al. 2015)
- C_{110} = 5.3 ± 1.0 mg/m^3 = airborne concentration of silver in the 110 nm AgNP aerosol (Anderson et al. 2015)
- T = 360 min = experimental exposure period (60 min/h, 6 h/day)
- V = 0.15 L/min = minute ventilation for a 300 g rat
- 10.4 cm^2 = SA of rat nasal cavity (RNC) (Derelanko and Auletta 2014)

Calculation of estimated silver deposition normalized to surface area of the rat nasal cavity after a single 6 h exposure to aerosolized AgNP with a 30 nm hydrodynamic diameter:

$$11\% \times \frac{7.2 \text{ mg Ag NP}}{\text{m}^3} \times \frac{\text{m}^3}{1000 \text{ L}} \times 360 \text{ min} \times \frac{0.15 \text{ L}}{\text{min}} = 0.04 \text{ mg AgNP}$$

$$\frac{0.04 \text{ mg Ag NP}}{10.4 \text{ cm}^2 \text{ RNC}} = \frac{0.004 \text{ mg Ag NP}}{\text{cm}^2 \text{ RNC}}$$

Calculation of estimated silver deposition normalized to surface area of the rat nasal cavity after a single 6 h exposure to aerosolized AgNP with a 110 nm diameter:

$$4\% \times \frac{5.3\ \text{mg Ag NP}}{\text{m}^3} \times \frac{\text{m}^3}{1000\ \text{L}} \times 360\ \text{min} \times \frac{0.15\ \text{L}}{\text{min}} = 0.01\ \text{mg AgNP}$$

$$\frac{0.01\ \text{mg Ag NP}}{10.4\ \text{cm}^2\ \text{RNC}} = \frac{0.001\ \text{mg Ag NP}}{\text{cm}^2\ \text{RNC}}$$

Table S1. Raw data on numbers of activated microglia to total microglia in control and AgNP-treated animals

Time point	Activated Microglia	Resting Microglia	% of Activated Microglia
Citrate Buffer	21	29	42
Citrate Buffer	33	17	66
Citrate Buffer	14	36	28
Citrate Buffer	19	31	38
Citrate Buffer	26	24	52
Citrate Buffer	30	20	60
20 nm AgNP T0	34	16	68
20 nm AgNP T0	36	14	72
20 nm AgNP T0	22	28	44
20 nm AgNP T1	39	11	78
20 nm AgNP T1	47	3	94
20 nm AgNP T1	31	19	62
20 nm AgNP T7	35	15	70
20 nm AgNP T7	33	17	66
20 nm AgNP T7	38	12	76
20 nm AgNP T21	32	18	64
20 nm AgNP T21	37	13	74
20 nm AgNP T21	25	25	50
20 nm AgNP T56	16	34	32
20 nm AgNP T56	34	16	68
20 nm AgNP T56	24	26	48
110 nm AgNP T0	38	12	76
110 nm AgNP T0	34	16	68
110 nm AgNP T0	32	18	64
110 nm AgNP T1	38	12	76
110 nm AgNP T1	26	24	52
110 nm AgNP T1	16	34	32
110 nm AgNP T7	31	19	62
110 nm AgNP T7	29	21	58
110 nm AgNP T7	19	31	38
110 nm AgNP T21	37	13	74
110 nm AgNP T21	32	18	64
110 nm AgNP T21	32	18	64
110 nm AgNP T56	32	18	64
110 nm AgNP T56	33	17	66

Table S2. Calculations of highest silver transport from the nose to the olfactory bulb at T0 for 20 nm and at T56 for 110 nm[a]

Particle type	Silver deposition in olfactory region of nose (μg Ag/g tissue)	Percentage of silver translocation to olfactory bulb from olfactory region of nose (%)
**20 nm AgNP**	(0.03408 μg Ag/g tissue in nose) x 0.2455 = 0.0837	(0.0132 μg Ag/g tissue in OB/0.0837 μg Ag/g tissue in olfactory region of nose) x 100 = 15.77% *at T0*
**110 nm AgNP**	(0.4312 μg Ag/g tissue in nose) x 0.35 = 0.1509	(0.0142 μg Ag/g tissue in OB/0.1509 μg Ag/g tissue in olfactory region of nose) x 100 = 9.41% *at T56*

[a]Calculations derived from Garcia and Kimbell (2009).

References

Anderson DS, Patchin ES, Silva RM, Uyeminami DL, Sharmah A, Guo T, et al. 2015. Influence of particle size on persistence and clearance of aerosolized silver nanoparticles in the rat lung. Toxicol Sci 1-16.

Cheng KH, Swift DL. 1995. Calculation of total deposition fraction of ultrafine aerosols in human extrathoracic and intrathoracic regions. Aerosol Sci Technol 22:194-201.

Derelanko MJ, Auletta CS. 2014. Handbook of Toxicology. 3rd ed. Boca Raton, FL: CRC Press.

Lee JH, Ahn K, Kim SM, Jeon KS, Lee JS, Yu IJ. 2012. Continuous 3-day exposure assessment of workplace manufacturing silver nanoparticles. J Nanopart Res 14:1134.

Stence N, Waite M, Dailey ME. 2001. Dynamics of microglial activation: a confocal time-lapse analysis in hippocampal slices. Glia 33:256–266.
